# Longitudinal patterns of cortical thinning in multiple sclerosis

**DOI:** 10.1002/hbm.24940

**Published:** 2020-02-17

**Authors:** Charidimos Tsagkas, M. Mallar Chakravarty, Laura Gaetano, Yvonne Naegelin, Michael Amann, Katrin Parmar, Athina Papadopoulou, Jens Wuerfel, Ludwig Kappos, Till Sprenger, Stefano Magon

**Affiliations:** ^1^ Neurologic Clinic and Policlinic, Departments of Medicine, Clinical Research and Biomedical Engineering University Hospital Basel and University of Basel Switzerland; ^2^ Translational Imaging in Neurology (ThINk) Basel, Department of Medicine and Biomedical Engineering University Hospital Basel and University of Basel Basel Switzerland; ^3^ Medical Image Analysis Center AG Basel Switzerland; ^4^ Cerebral Imaging Centre – Douglas Mental Health University Institute Verdun, QC Canada; ^5^ Department of Biomedical Engineering McGill University Montreal, QC Canada; ^6^ Department of Psychiatry McGill University Montreal, QC Canada; ^7^ F. Hoffmann‐La Roche Ltd Basel Switzerland; ^8^ Department of Biomedical Engineering University of Basel Allschwil Switzerland; ^9^ NeuroCure Clinical Research Center, Charite ‐ Universitatsmedizin Berlin, corporate member of Freie Universitat Berlin, Humboldt‐Universitat zu Berlin, and Berlin Institute of Health Berlin Germany; ^10^ Department of Neurology DKD HELIOS Klinik Wiesbaden Wiesbaden Germany; ^11^ Roche Pharma Research and Early Development Roche Innovation Center Basel, F. Hoffmann‐La Roche Ltd. Basel Switzerland

**Keywords:** atrophy, biomarkers, cortical thickness, MRI, multiple sclerosis, T2 lesions

## Abstract

In multiple sclerosis (MS), cortical atrophy is correlated with clinical and neuropsychological measures. We aimed to examine the differences in the temporospatial evolution of cortical thickness (CTh) between MS‐subtypes and to study the association of CTh with T2‐weighted white matter lesions (T2LV) and clinical progression. Two hundred and forty‐three MS patients (180 relapsing–remitting [RRMS], 51 secondary‐progressive [SPMS], and 12 primary‐progressive [PPMS]) underwent annual clinical (incl. expanded disability status scale [EDSS]) and MRI‐examinations over 6 years. T2LV and CTh were measured. CTh did not differ between MS‐subgroups. Higher total T2LV was associated with extended bilateral CTh‐reduction on average, but did not correlate with CTh‐changes over time. In RRMS, CTh‐ and EDSS‐changes over time were negatively correlated in large bilateral prefrontal, frontal, parietal, temporal, and occipital areas. In SPMS, CTh was not associated with the EDSS. In PPMS, CTh‐ and EDSS‐changes over time were correlated in small clusters predominantly in left parietal areas. Increase of brain lesion load does not lead to an immediate CTh‐reduction. Although CTh did not differ between MS‐subtypes, a dissociation in the correlation between CTh‐ and EDSS‐changes over time between RRMS and progressive‐MS was shown, possibly underlining the contribution of subcortical pathology to clinical progression in progressive‐MS.

**Abbreviations**
CTh: cortical thickness
EDSS: Expanded Disability Status Scale
GM: gray matter
PASAT: Paced Auditory Serial Addition Test
PPMS: primary progressive multiple sclerosis
RRMS: relapsing remitting multiple sclerosis
SDMT: Symbol Digit Modalities Test
SPMS: secondary progressive multiple sclerosis
T25fwt: timed 25‐ft walk test
T2LV: T2 lesion volume
WM: white matter

## 1 INTRODUCTION

MS is a chronic inflammatory, demyelinating and neurodegenerative disease of the CNS, affecting more than 2 million people worldwide (GBD 2015 Neurological Disorders Collaborator Group, 2017). In the past decade, cortical gray matter (GM) pathology has been established as an important contributing mechanism in this debilitating disorder (Fisher, Lee, Nakamura, & Rudick, 2008; Geurts & Barkhof, 2008; Roosendaal et al., 2011). GM atrophy can be quantified in an automated fashion in vivo using MRI (Amiri et al., 2018) and most likely reflects a diffuse reduction in cortical neuronal density, axonal density, and neuronal size (Popescu et al., 2015). This process has been shown to be associated with cortical lesion volume (Calabrese et al., 2010) and is thought to be, at least in part, the consequence of a pathogenic process driven from pial lesions (Mainero et al., 2015). Cross‐sectional analyses point to an additional contribution of demyelination in white matter (WM) tracts connecting the brain cortex with subcortical structures (e.g., deep GM) to this disease component (Kolasinski et al., 2012; Steenwijk et al., 2014); however, currently there is a lack of longitudinal data supporting this pathomechanism.

Cortical GM atrophy is prevalent in all MS subtypes as early as at the stage of clinically isolated syndromes and becomes more widespread and severe with increasing disease duration (Eshaghi et al., 2018; Fisher et al., 2008; Roosendaal et al., 2011). Despite the fact that whole GM volume loss does not differ between MS disease subtypes, a recent multicenter study showed accelerated GM atrophy rates in the temporal lobe in secondary progressive (SPMS) compared to relapsing remitting MS (RRMS) (Eshaghi et al., 2018). This suggests that, the rate of GM volume loss in the temporal lobe may be a surrogate for transition from the RRMS to the SPMS phase of the disease. However, it is unclear if this observed between‐group difference concerns a specific temporal lobe GM region or diffuse temporal cortical volume loss and whether the observed between MS disease subtype difference may be ascribed to the higher age of the SPMS patients.

Increasing evidence from both cross‐sectional and longitudinal analyses points towards an association of cortical GM pathology such as cortical lesions and atrophy with physical and especially with cognitive dysfunction (Bergsland et al., 2017; Eijlers et al., 2018; Fisniku et al., 2008). However, the exact anatomical substrate of the cortical GM loss driving disease progression is still poorly understood.

In this study, we examined the cortical thickness (CTh) changes in a large cohort of MS patients over 6 years. We aimed at localizing differences in the reduction of cortical GM between disease phenotypes and to study the contribution of CTh changes to the progression of physical and cognitive disability progression. We also examined the effect of WM lesions on cortical GM changes over time.

## 2 MATERIALS AND METHODS

### 2.1 Study design and participants

We analyzed clinical and MRI data of RRMS, SPMS, and primary progressive (PPMS) patients (Table 1) from an ongoing large scale cohort study (GeneMSA) at a single center (Multiple Sclerosis Center, University Hospital, Basel, Switzerland) (Tsagkas et al., 2018; Tsagkas et al., 2019). Patients were followed over a maximum of 6 years (7 annual time points). Both clinical assessments and the MRI examinations were performed at least 1 month after the occurrence of a clinical relapse or treatment with glucocorticosteroids. The diagnosis of MS was made in accordance with international panel established criteria (McDonald et al., 2001). The study was approved by the local ethics committee. All patients included in this work have been previously reported in former studies (Tsagkas et al., 2018; Tsagkas et al., 2019).

**Table 1:** Demographics and clinical characteristics of patients with MS

Characteristics	Overall	RRMS	SPMS	PPMS	*p*‐value
Number of patients	231	180	51	12	
Baseline age (y)					*** ‡
*Mean (SD)*	44.5 (11.1)	41.4 (10.2)	55.3 (7.6)	46.42 (6.6)	
Sex (female/male)	166/77	133/47	27/24	6/6	*
Baseline disease duration (y)					*** ‡‡‡
*Mean (SD)*	12.7 (9.1)	11.2 (8.3)	19.0 (9.7)	8.33 (7.1)	
Baseline EDSS					*** †††
*Median (SD)*	3.0 (1.7)	2.5 (1.4)	4.5 (1.4)	4.25 (1.5)	
Annual EDSS change					*
*Mean (SD)*	0.12 (0.35)	0.10 (0.33)	0.22 (0.41)	0.11 (0.15)	
Baseline T25fwt (s)					*** †
*Mean (SD)*	7.55 (10.60)	5.68 (7.08)	14.15 (17.39)	8.73 (7.27)	
Annual T25fwt change					*** †
*Mean (SD)*	1.01 (4.33)	0.17 (1.94)	4.12 (8.25)	1.51 (2.44)	
Baseline PASAT					**
*Mean (SD)*	43.75 (11.7)	44.9 (11.4)	39.3 (12.1)	44.8 (11.6)	
Annual PASAT change					n.s.
*Mean (SD)*	0.34 (4.23)	0.38 (3.86)	0.40 (5.61)	−0.48 (2.61)	
Baseline SDMT					**
*Mean ± SD*	43.75 (11.7)	48.5 (13.7)	40.9 (9.8)	43.6 (6.6)	
Annual SDMT change					n.s.
*Mean (SD)*	0.68 (2.75)	0.91 (2.69)	0.14 (3.16)	−0.31 (0.97)	
ARR					** ††
*Mean (SD)*	0.32 (0.48)	0.39 (0.52)	0.16 (0.30)	0 (0)	
Baseline treatment					††† ‡‡‡
*Untreated*	87	58	17	12	
*Azathioprine*	6	4	2	0	
*Interferon*	117	91	26	0	
*Glatimer acetate*	29	26	3	0	
*Mitoxantrone*	4	1	3	0	
Number of follow‐ups					n.s.
*Mean ± SD*	5.11 (1.96)	5.16 (1.99)	4.88 (2.00)	5.25 (1.48)	
Maximum follow‐up time					n.s.
*Mean (SD)*	4.36 (2.03)	4.41 (2.05)	4.11 (2.06)	4.67 (1.67)	

Abbreviations: ARR, annualized relapse rate; EDSS, Expanded Disability Status Scale; PPMS, primary progressive multiple sclerosis; T25fwt, timed 25‐foot walk test; RRMS, relapsing remitting multiple sclerosis; SPMS, secondary progressive multiple sclerosis.

*Note:* n.s., not significant for any comparisons between PPMS, RRMS, and SPMS.

RRMS versus SPMS: * ≤ .05, ** ≤ .01, *** ≤ .001.

RRMS versus PPMS: † ≤ .05, †† ≤ .01, ††† ≤ .001.

PPMS versus SPMS: ‡ ≤ .05, ‡‡ ≤ .01, ‡‡‡ ≤ .001.

Between‐group comparisons were performed using Welch's two sample *t* test and Pearson's chi‐squared test with Yate's continuity correction where appropriate.

### 2.2 Clinical assessment

All patients underwent a standardized neurological examination including the Expanded Disability Status Scale (EDSS; http://www.neurostatus.org) by trained and certified examiners, Timed 25‐foot walk test (T25fwt) and Paced Auditory Serial Addition Test (PASAT) annually. Patients also underwent an annual Symbol Digit Modalities Test (SDMT) starting at the fourth follow‐up time (or at the third year of monitoring). No parallel test versions were used for the SDMT, whereas two versions of the PASAT were deployed for annual neuropsychological tests. Relapses that occurred 12 months prior to each follow‐up were also recorded. All clinical and neuropsychological metrics were recorded as cross‐sectional measurements at each follow‐up, whereas longitudinal changes of those metrics were not documented prospectively.

### 2.3 MRI protocol

Morphological analyses were performed on high‐resolution three‐dimensional T1w MPRAGE images acquired in sagittal plane (TR/TI/TE = 2080/1100/3.0 ms; *α* = 15°, 160 slices, voxel size: 0.98 × 0.98 × 1 mm). Additionally, a double spin echo proton density‐weighted (PD)/T2‐weighted sequence was acquired (TR/TE1/TE2 = 3980/14/108 ms; flip angle = 180°, 40 slices, 3 mm slice thickness without gap with an in‐plane resolution of 1mm^2^). All MRI scans were performed on a 1.5 Tesla Magnetom Avanto MRI‐scanner (Siemens Medical Solutions, Erlangen, Germany).

### 2.4 MRI analysis

All brain WM lesions were segmented on the PD‐weighted images by trained expert observers according to the standard operating procedures used at the local institution for the analysis of clinical phase II and phase III trial. T2‐weighted lesion volume (T2LV) was calculated for the whole brain as well as for each lobe as segmented by the “Automatic Nonlinear Image Matching and Anatomical Labeling” algorithm (ANIMAL) (Collins, Holmes, Peters, & Evans, 1995) at all available time points.

In order to avoid misclassification of lesions as GM, lesions masks generated on PD‐weighted images were used to fill the lesions on T1‐weighted images with the intensity of the surrounding white matter tissue (Magon et al., 2014). CTh was estimated on the lesion‐filled T1‐weighted images using the fully automated CIVET 1.1.10 pipeline (Collins, Neelin, Peters, & Evans, 1994; Lyttelton, Boucher, Robbins, & Evans, 2007). Summarizing this process, the T1‐weighted images were linearly registered to the standard stereotaxic space defined by the MNI ICBM 152 model (Mazziotta et al., 2001). The images were then corrected for intensity nonuniformity using N3 (Sled, Zijdenbos, & Evans, 1998) and a nonlinear registration to the model (Collins et al., 1994) was applied. The tissue classification was performed using INSECT, whose output was then fed to a Partial Volume Estimator, which in turn is used for the actual surface fitting (Tohka, Zijdenbos, & Evans, 2004). Each voxel was classified as WM, GM, or CSF. The images were then mapped to a probabilistic atlas using the ANIMAL algorithm. Finally, the WM surface was generated by using a deformable ellipsoid polygonal model that shrinks until it fits the WM/GM interface. To generate the GM surface, the WM surface was expanded until the GM/CSF interface (or pial surface) is reached using a Laplacian approach in order to find the best fit (Jones, Buchbinder, & Aharon, 2000; Kim et al., 2005). Specifically, to adequately estimate the CTh, the Laplace's equation describes a smooth trajectory between the WM and GM surfaces defining a layered set of surfaces (Jones et al., 2000). Thus, each vertex on the WM surface maps to a specific point in the GM surface and back to the same point in WM surface. The CTh is estimated as the distance, in millimeters, between WM and GM surfaces at each vertex. The surfaces are composed of 40,962 vertices for each hemisphere. After statistical analysis, we used the Automated Anatomical Labeling (AAL) atlas to determine the localization of the identified significant clusters (Ad‐Dab'bagh et al., 2006; Lyttelton et al., 2007; Tzourio‐Mazoyer et al., 2002). Based on this atlas, our results are reported in the form of mean *t*‐value (MTV) ± *SD* and number of vertexes of each cluster in the individual cortical regions.

### 2.5 Statistical analysis

Comparisons of demographic factors, clinical measurements, and number of follow‐ups between MS subtypes were made using Welch's and Pearson's chi‐squared test with Yate's continuity correction. A logarithmic transformation of the EDSS was performed in order to correct for its nonlinearity in representing physical disability, as conducted in previous studies (Magon et al., 2014; Tsagkas et al., 2018; Tsagkas et al., 2019). The annualized relapse rate was calculated for each patient.

Vertex‐wise longitudinal analysis was performed using a linear mixed effect model (LMER) in order to explore longitudinal correlations between the patients' CTh and demographic, clinical and T2LV measurements. LMER was also used to examine the trends of PASAT and SDMT changes over time in our cohort after a square transformation for PASAT in order to approximate a normal distribution. This was done using a random intercept and a random time slope for each subject to allow for within‐subject and between‐subject variance. CTh was always used as the dependent variable in our analysis. For the investigation of the association between CTh and clinical outcomes or T2LV, the independent variables were entered blockwise keeping the following sequence: first demographics and then clinical variables or T2LV respectively. Separate analyses were conducted for the whole brain T2LV as well as for the left and right T2LV of the frontal, parietal, temporal, and occipital WM. Each variable was tested both for its correlation to the CTh intercept as well as to the CTh slope over time. All independent variables without statistical significance were excluded from the final model. In order to reduce the risk of type I errors the results were corrected for multiple comparisons by using the False Discovery Rate approach set at *q* < 0.05.

In order to assess between‐group CTh differences of RRMS and SPMS with PPMS patients, we performed propensity‐score matching baseline covariates, including sex, age and disease duration as described in a previous study (Tsagkas et al., 2019). RRMS and SPMS were matched with PPMS patients, based on high similarity of propensity scores, on a 2:1 basis for each group and all groups had a similar follow‐up time. Comparisons of the RRMS and SPMS CTh with PPMS were done using vertex‐wise LMER using the False Discovery Rate approach set at *q* < 0.01 (instead of 0.05) in order to correct for multiple comparisons between the patient groups.

Beside the multiple comparison correction approaches discussed above, no other approach was used in the rest of the analysis, since the models used for the examination of the correlation between CTh and demographical/clinical data are independent from each other.

All statistical analyses of CTh were performed in R (https://www.r-project.org/) using the RMINC package (https://wiki.mouseimaging.ca/display/MICePub/RMINC).

## 3 RESULTS

A total of 243 MS patients (180 RRMS, 51 SPMS, and 12 PPMS) were monitored yearly over an average time span of 4.36 ± 2.03 years. Ninety‐three patients, completed all 7 scans, whereas another 35 completed 6 scans and 29 completed 5 scans. The rest of the patients (86) completed four scans or less. Demographics and clinical characteristics of our cohort are described in Table 1.

### 3.1 Reduction of cortical thickness over time

**Figure 1:**
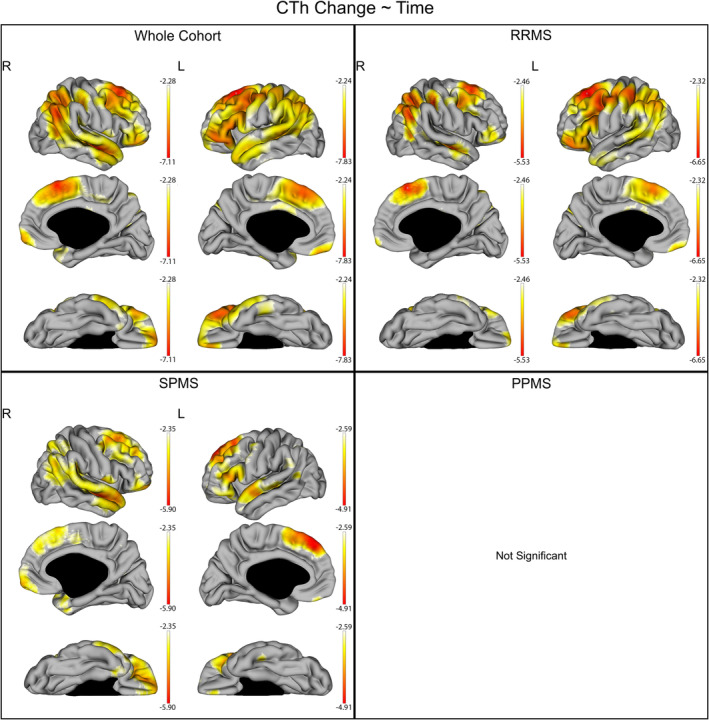
CTh reduction over time in the whole cohort and individual subgroups of disease subtypes. The gradient from yellow to red indicates a lower to higher negative reduction respectively, as shown by the *t*‐values extracted from our linear mixed effect models. In each graph, the highest (or less negative) gradient value represents the threshold of the respective *t*‐values after correction with the false discovery rate approach for multiple comparisons set at q < 0.05. Up left: CTh reduction over time in the whole cohort. Up right: CTh reduction over time in the relapsing remitting multiple sclerosis (RRMS). Down left: CTh reduction over time in the secondary progressive multiple sclerosis (SPMS). Down right: no statistically significant CTh reduction over time was shown in the primary progressive multiple sclerosis (PPMS)

Reduction of CTh in the whole cohort and each individual MS subtypes are graphically displayed in Figure 1. In the whole cohort as well as in RRMS and SPMS patients separately, cortical thickness reduced in extended cortical regions predominantly in the prefrontal, frontal, parietal and temporal lobes (overall MTV; whole cohort: right −3.72 ± 0.97, left −3.92 ± 1.02; RRMS: right −3.41 ± 0.69, left −3.51 ± 0.85; SPMS: right −3.11 ± 0.56, left −3.31 ± 0.52). No significant CTh reduction was found in the PPMS group (*n* = 12).

### 3.2 Association of CTh with demographic factors

**Figure 2:**
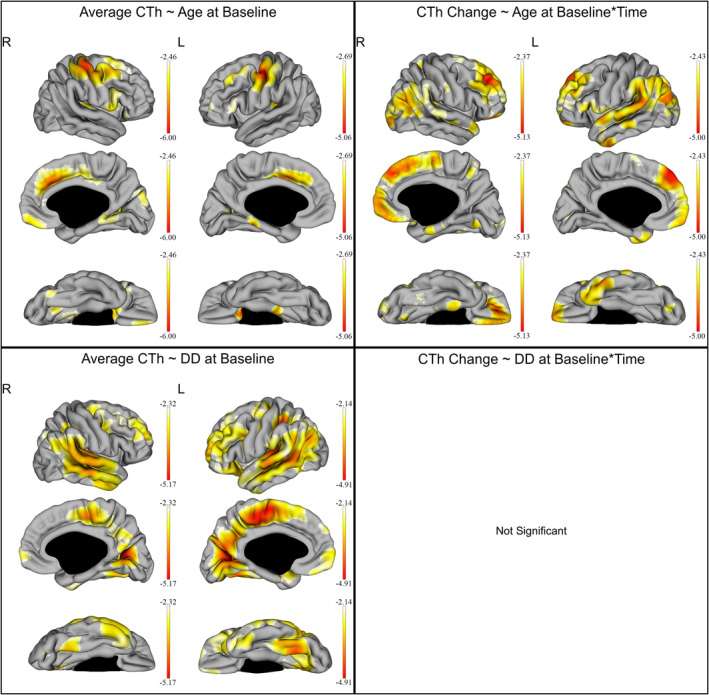
Effect of age and disease duration (DD) on the cortical thickness (CTh) of all multiple sclerosis patients. The gradient from yellow to red indicates a weaker to stronger negative correlation respectively, as shown by the *t*‐values extracted from our linear mixed effect models. In each graph, the highest (or less negative) gradient value represents the threshold of the respective *t*‐values after correction with the false discovery rate approach for multiple comparisons set at *q* < 0.05. Up left: correlation of the average CTh with age at baseline. Up right: correlation of the CTh changes over time with age at baseline. Down left: correlation of the average CTh with DD at baseline. Down right: no statistically significant correlation was shown between CTh changes over time and DD at baseline

Associations between demographic factors and CTh are graphically displayed in Figure 2 and Table 2. Age at baseline was associated with a reduction of the average CTh predominantly in the parietal, prefrontral, and frontal cortex bilaterally, while being slightly more extended in the right hemisphere (*q* < 0.05). Age at baseline was also negatively associated with CTh changes over time in extended cortical regions mostly involving the bilateral prefrontal cortex, bilateral parieto‐occipital regions, and the superior temporal gyri (*q* < 0.05). Disease duration at baseline was also associated with extended cortical thinning of the bilateral frontal and prefrontal cortex as well as large parietal, temporal, and occipital CTh reduction—more extended in the left hemisphere, but was not correlated with the CTh changes over time (*q* < 0.05). Sex was not correlated with CTh or its changes over time. In RRMS, the annualized relapse rate was not associated with CTh or its changes over time.

**Table 2:** Association of average cortical thickness and cortical thickness changes over time with age and disease duration at baseline

Cortical regions	Gyri	Age at baseline—average CTh		Age at baseline—CTh changes over time		DD at baseline—average CTh		DD at baseline—CTh changes over time	
		Right	Left	Right	Left	Right	Left	Right	Left
Central region	Precentral gyrus	−3.88 (0.86), 974	−3.78 (0.61), 686	−2.48 (0.12), 6	−2.57 (0.06), 15	−2.78 (0.30), 273	−3.13 (0.63), 752	—	—
	Postcentral gyrus	−3.48 (0.55), 737	−3.40 (0.42), 578	−2.67 (0.23), 121	−2.59 (0.12), 53	−2.69 (0.29), 410	−3.07 (0.66), 799	—	—
	Rolandic operculum	−2.95 (0.28), 164	−2.83 (0.08), 36	−2.87 (0.24), 143	−2.87 (0.20), 275	−2.44 (0.10), 65	−3.04 (0.59), 106	—	—
Frontal lobe	Superior frontal gyrus, dorsolateral	−2.81 (0.86), 339	−2.76 (0.06), 10	−3.13 (0.55), 887	−3.12 (0.08), 981	−2.75 (0.27), 779	−2.70 (0.38), 1,201	—	—
	Superior frontal gyrus, medial	−2.86 (0.33), 145	−3.18 (0.26), 278	−3.40 (0.55), 695	−3.33 (0.73), 841	−2.51 (0.13), 78	−2.55 (0.26), 346	—	—
	Superior frontal gyrus, orbital part	−2.90 (0.32), 35	−3.10 (0.26), 37	−3.40 (0.68), 476	−3.07 (0.44), 430	−2.41 (0.05), 13	−2.69 (0.32), 276	—	—
	Superior frontal gyrus), medial orbital	−2.53 (0.05), 8	—	−3.22 (0.47), 279	−3.04 (0.35), 191	−2.59 (0.15), 116	−2.56 (0.21), 215	—	—
	Middle frontal gyrus	−3.27 (0.45), 560	−2.96 (0.20), 469	−3.49 (0.73), 1,244	−3.08 (0.38), 929	−2.70 (0.23), 1,489	−2.68 (0.30), 1,468	—	—
	Middle frontal gyrus orbital part	—	−2.80 (0.05), 14	−3.10 (0.46), 96	−2.54 (0.45), 13	—	−2.33 (0.15), 147	—	—
	Inferior frontal gyrus, opercular part	−3.18 (0.32), 467	−2.92 (0.15), 77	−2.55 (0.12), 98	−2.89 (0.06), 252	−2.70 (0.21), 82	−2.78 (0.36), 123	—	—
	Inferior frontal gyrus, triangular part	−2.78 (0.22), 320	−2.82 (0.12), 208	−2.75 (0.26), 292	−2.94 (0.27), 307	−2.50 (0.13), 89	−2.62 (0.32), 427	—	—
	Inferior frontal gyrus, orbital part	−2.84 (0.46), 155	—	−2.68 (0.31), 164	—	—	−2.74 (0.37), 854	—	—
	Supplementary motor area	−2.92 (0.31), 365	−3.40 (0.50), 273	−2.95 (0.40), 584	−2.51 (0.07), 40	−3.17 (0.51), 560	−3.29 (0.74), 733	—	—
	Paracentral lobule	−2.53 (0.05), 3	—	−2.45 (0.11), 3	—	−2.84 (0.28), 289	−3.51 (0.81), 635	—	—
	Gyrus rectus	2.94 (0.30), 178	−2.96 (0.20), 27	−3.29 (0.42), 344	−3.04 (0.39), 151	−2.44 (0.07), 19	−2.36 (0.14), 176	—	—
	Olfactory cortex	−3.52 (0.57), 83	−3.86 (0.50), 92	−2.54 (0.12), 7	—	—	—	—	—
Temporal lobe	Heschl gyrus	−3.07 (0.40), 226	−3.05 (0.20), 179	−2.75 (0.26), 98	−2.70 (0.16), 64	−3.59 (0.42), 245	−3.91 (0.60), 271	—	—
	Superior temporal gyrus	−3.02 (0.58), 332	−2.95 (0.16), 223	−2.65 (0.20), 678	−3.22 (0.46), 1,230	−3.39 (0.43), 1,612	−3.00 (0.58), 1,211	—	—
	Middle temporal gyrus	—	—	−2.94 (0.45), 785	−2.84 (0.28), 1,175	−3.11 (0.45), 1,578	−3.20 (0.51), 1962	—	—
	Inferior temporal gyrus	—	—	−2.52 (0.14), 10	−3.21 (0.44), 307	−2.96 (0.33), 466	−2.55 (0.25), 322	—	—
Parietal lobe	Superior parietal gyrus	−2.75 (0.20), 47	—	−2.63 (0.24), 65	−2.72 (0.17), 111	−2.90 (0.30), 442	−2.66 (0.62), 536	—	—
	Inferior parietal, but supramarginal and angular gyri	—	—	−2.60 (0.18), 52	−2.87 (0.29), 302	−2.89 (0.30), 242	−2.74 (0.53), 467	—	—
	Angular gyrus	—	—	−2.87 (0.29), 344	−3.07 (0.31), 572	−2.91 (0.29), 331	−2.82 (0.47), 506	—	—
	Supramarginal gyrus	−2.54 (0.07), 18	−2.81 (0.07), 12	−2.66 (0.21), 370	−3.09 (0.36), 414	−3.30 (0.55), 601	−2.94 (0.58), 513	—	—
	Precuneus	−2.78 (0.20), 323	−2.85 (0.10), 29	−2.61 (0.23), 236	−2.49 (0.04), 11	−2.74 (0.25), 1,008	−3.24 (0.64), 1,451	—	—
Occipital lobe	Superior occipital gyrus	—	—	−2.74 (0.22), 325	−2.79 (0.26), 219	−2.84 (0.31), 209	−2.42 (0.12), 502	—	—
	Middle occipital gyrus	—	—	−3.10 (0.43), 853	−3.14 (0.48), 1,014	−2.60 (0.25), 111	−2.85 (0.39), 1,238	—	—
	Inferior occipital gyrus	−2.75 (0.20), 42	—	−3.24 (0.53), 100	−2.70 (0.12), 22	−2.47 (0.09), 16	−2.80 (0.36), 199	—	—
	Cuneus	−2.85 (0.25), 624	−2.98 (0.17), 80	—	—	−3.38 (0.78), 514	−3.62 (0.83), 1,103	—	—
	Calcarine fissure and surrounding cortex	−2.74 (0.20), 148	−2.89 (0.14), 32	−2.63 (0.17), 163	−2.60 (0.13), 67	−3.44 (0.72), 482	−3.44 (0.73), 598	—	—
	Lingual gyrus	−2.84 (0.19), 349	−2.99 (0.22), 47	−2.85 (0.32), 80	−2.89 (0.22), 98	−2.77 (0.39), 264	−2.91 (0.56), 672	—	—
	Fusiform gyrus	−2.70 (0.15), 29	—	−2.49 (0.09), 41	−3.11 (0.33), 41	−2.89 (0.37), 497	−3.01 (0.53), 824	—	—
Limbic lobe	Temporal pole: Superior temporal gyrus	−2.82 (0.25), 85	—	−2.74 (0.27), 257	−2.81 (0.26), 140	−2.55 (0.16), 108	−2.64 (0.28), 505	—	—
	Temporal pole: Middle temporal gyrus	—	—	−2.65 (0.20), 71	−3.24 (0.29), 118	−2.60 (0.20), 108	−2.33 (0.14), 79	—	—
	Anterior cingulate and paracingulate gyri	−3.38 (0.66), 452	−2.88 (0.21), 13	−3.07 (0.61), 133	—	—	−2.18 (0.06), 2	—	—
	Median cingulate and paracingulate gyri	−3.33 (0.65), 669	−3.42 (0.44), 280	−3.35 (0.54), 118	−2.48 (NA), 1	−2.65 (0.22), 227	−2.84 (0.46), 442	—	—
	Posterior cingulate gyrus	—	—	—	—	−2.55 (0.11), 72	−3.28 (0.50), 153	—	—
	Parahippocampal gyrus	−2.90 (0.31), 193	−3.32 (0.36), 154	−2.76 (0.29), 142	−2.78 (0.29), 7	−2.67 (0.18), 72	−2.31 (0.15), 157	—	—
Insular lobe	Insula	−2.78 (0.24), 227	−2.95 (0.17), 87	−2.61 (0.17), 92	−2.59 (0.09), 26	−2.52 (0.14), 39	−2.70 (0.42), 381	—	—

*Note:* Effect of age and disease duration (DD) on the cortical thickness (CTh) of significantly associated cortical regions in all multiple sclerosis patients. Results represent the correlation strength shown in the form of *t*‐values extracted from our linear mixed effect models after correction with the false discovery rate approach for multiple comparisons set at *q* < 0.05. Lower (or more negative) *t*‐values reflect a stronger negative correlation. All results are reported in the form: mean *t*‐value (*SD*), number of vertexes in the respective region.

Abbreviation: NA, not applicable.

### 3.3 CTh differences between groups

CTh and its changes over time did not differ between RRMS and SPMS patients after correcting for age and disease duration at baseline. In sex‐, age‐ and disease duration‐matched subgroups of 60 RRMS, SPMS, and PPMS patients (24 RRMS, 24 SPMS and 12 PPMS, mean age at baseline 48.9 ± 8.2 years, mean disease duration 10.2 ± 6.6 years, 32 female), also no differences in CTh and its changes over time were found among groups.

### 3.4 Association of CTh with WM lesion load

**Figure 3:**
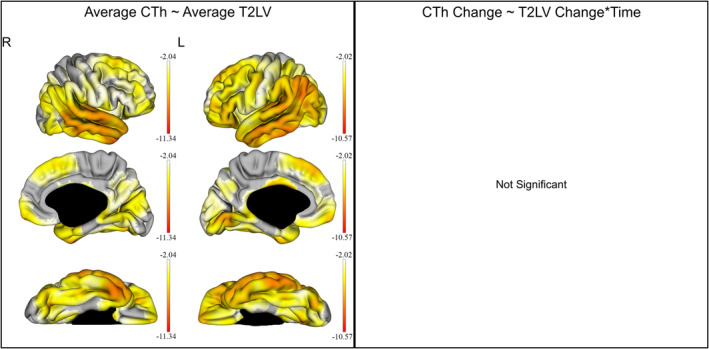
Effect of the whole brain T2w‐lesion (T2LV) on the cortical thickness (CTh) of all multiple sclerosis patients. The gradient from yellow to red indicates a weaker to stronger negative correlation respectively, as shown by the *t*‐values extracted from our linear mixed effect models. In each graph, the highest (or less negative) gradient value represents the threshold of the respective *t*‐values after correction with the false discovery rate approach for multiple comparisons set at *q* < 0.05. Left: correlation of the average CTh with the average T2LV. Right: no statistically significant correlation was shown between CTh and T2LV changes over time

After correcting for age and disease duration, a negative correlation was found between whole brain T2LV and the average CTh in regions extending symmetrically in nearly the whole cortex bilaterally (overall right: MTV −4.00 ± 1.36; left: MTV −4.24 ± 1.35, *q* < 0.05). Whole brain T2LV changes were not associated with the CTh changes over time. These results are also graphically displayed in Figure 3.

**Figure 4:**
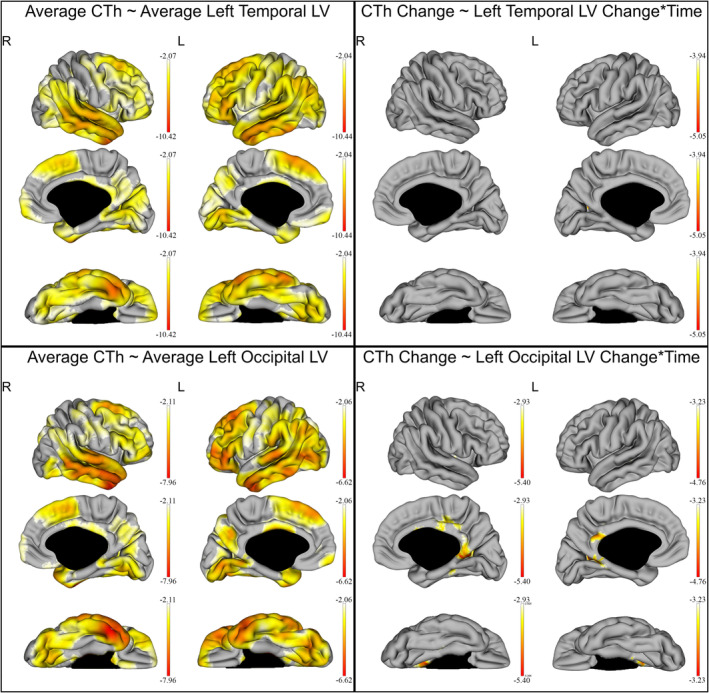
Effect of the left temporal and left occipital T2w‐lesion (T2LV) on the cortical thickness (CTh) of all multiple sclerosis patients. The gradient from yellow to red indicates a weaker to stronger negative correlation respectively, as shown by the *t*‐values extracted from our linear mixed effect models. In each graph, the highest (or less negative) gradient value represents the threshold of the respective *t*‐values after correction with the false discovery rate approach for multiple comparisons set at *q* < 0.05. Up left: correlation of the average CTh with the average left temporal T2LV. Up right: correlation of the CTh and left temporal T2LV changes over time. The right hemisphere was added in this figure solely for completion, since no correlation between CTh in the right hemisphere and left temporal T2LV changes over time was found. Down left: correlation of the average CTh with the average left occipital T2LV. Up right: correlation of the CTh and left occipital T2LV changes over time

When examining the relation between regional T2LV (left and right frontal, parietal, temporal and occipital WM lesions) and CTh, all regional T2LV were associated with a reduction of CTh in extended bilateral cortical regions, analogous to the whole brain T2LV (*q* < 0.05). In addition, left temporal T2LV changes were negatively correlated with the CTh change over time in a small cluster in the left cuneus (MTV −4.47 ± 0.35, 35 vertexes, *q* < 0.05), and precuneus (MTV −4.43 ± 0.29, 26 vertexes, *q* < 0.05). Left occipital T2LV changes were also negatively correlated with the CTh change over time predominantly in small bilateral temporal, parietal, and occipital regions (*q* < 0.05). The correlation between left temporal and occipital T2LV and CTh is shown in Figure 4, whereas the correlation between occipital T2LV and CTh is displayed in detail in Table 3.

**Table 3:** Association of cortical thickness changes over time with left occipital T2LV changes

Cortical regions	Gyri	MTV	
		Right	Left
Central region	Rolandic operculum	−3.09 (0.13), 25	—
Frontal lobe	Inferior frontal gyrus, opercular part	−3.24 (0.20), 34	—
	Supplementary motor area	−3.33 (0.25), 59	—
	Paracentral lobule	−3.19 (0.19), 24	—
Temporal lobe	Superior temporal gyrus	−3.09 (0.14), 14	−3.27 (0.46), 2
	Middle temporal gyrus	—	−3.33 (0.07), 27
Parietal lobe	Precuneus	−3.63 (0.46), 499	−3.97 (0.43), 242
Occipital lobe	Cuneus	−3.63 (0.46), 189	−3.97 (0.43), 175
	Calcarine fissure and surrounding cortex	−3.62 (0.50), 31	−3.48 (0.22), 7
	Lingual gyrus	−3.06 (0.09), 15	−3.62 (0.29), 63
	Fusiform gyrus	−3.82 (0.54), 96	—
Limbic lobe	Anterior cingulate and paracingulate gyri	−3.68 (0.52), 34	—
	Median cingulate and paracingulate gyri	−3.58 (0.43), 468	−3.47 (0.18), 106
	Posterior cingulate gyrus	−3.34 (0.32), 103	−3.74 (0.26), 68
	Parahippocampal gyrus	−3.58 (0.47), 67	−3.44 (0.16), 6
Insular lobe	Insula	−3.23 (0.22), 94	—

*Note:* Correlation between left occipital T2‐weighted Lesion Volume (T2LV) and the cortical thickness (CTh) of significantly associated cortical regions in all multiple sclerosis patients. Results represent the correlation strength shown in the form of *t*‐values extracted from our linear mixed effect models after correction with the false discovery rate approach for multiple comparisons set at *q* < 0.05. Lower (or more negative) *t*‐values reflect a stronger negative correlation. All results are reported in the form: mean *t*‐value (*SD*), number of vertexes in the respective region.

Abbreviation: NA, not applicable.

### 3.5 Association of CTh with the EDSS

#### 3.5.1 Whole cohort

**Figure 5:**
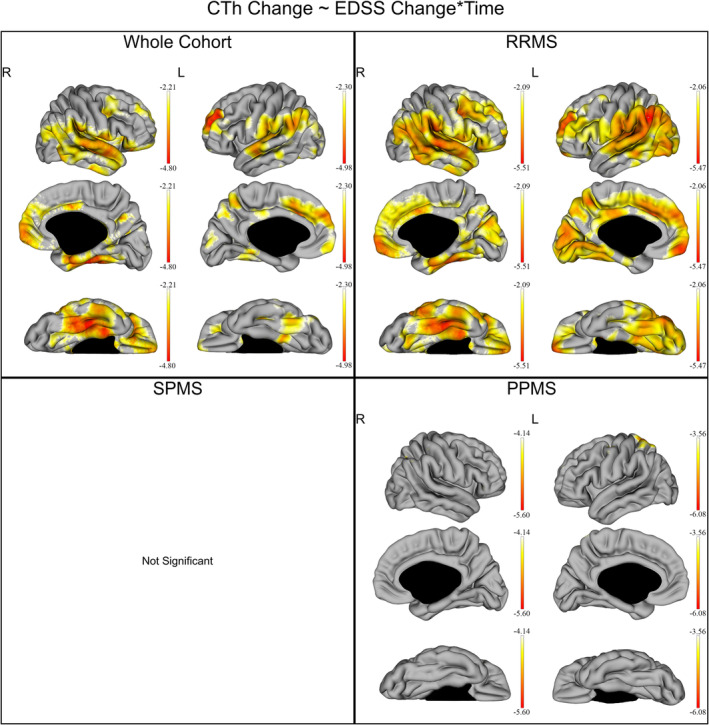
Correlation between EDSS and CTh changes over time in the whole cohort and individual subgroups of disease subtypes. The gradient from yellow to red indicates a weaker to stronger negative correlation respectively, as shown by the *t*‐values extracted from our linear mixed effect models. In each graph, the highest (or less negative) gradient value represents the threshold of the respective *t*‐values after correction with the false discovery rate approach for multiple comparisons set at *q* < 0.05. Up left: correlation between EDSS and CTh changes over time in the whole cohort. Up right: correlation between EDSS and CTh changes over time in the relapsing remitting multiple sclerosis (RRMS). Down left: no statistically significant correlation was shown between EDSS and CTh changes over time in the secondary progressive multiple sclerosis (SPMS). Down right: correlation between EDSS and CTh changes over time in the primary progressive multiple sclerosis (PPMS)

In the whole cohort, the log(EDSS) was not associated with the average CTh, after correcting for age and disease duration. However, log(EDSS) changes were negatively correlated with the CTh changes over time in large extended bilateral cortical regions (Figure 5), predominantly in the right temporal and left frontal and parietal lobes (*q* < 0.05). These results are also shown in detail in Table 4.

**Table 4:** Association of cortical thickness changes over time with EDSS changes by disease subtypes

Cortical region	Gyri	Overall		RRMS		SPMS		PPMS	
		Right	Left	Right	Left	Right	Left	Right	Left
Central region	Precentral gyrus	−2.66 (0.23), 92	−2.66 (0.23), 194	−2.79 (0.52), 417	−2.71 (0.30), 321	—	—	—	−4.42 (0.63), 89
	Postcentral gyrus	−3.05 (0.47), 189	−2.95 (0.44), 358	−2.94 (0.57), 394	−3.10 (0.56), 673	—	—	−4.24 (0.12), 2	−4.00 (0.42), 36
	Rolandic operculum	−3.39 (0.32), 456	−2.64 (0.22), 256	−3.78 (0.38), 456	−3.12 (0.50), 434	—	—	—	—
Frontal lobe	Superior frontal gyrus, dorsolateral	−2.54 (0.21), 418	−3.80 (0.73), 808	−2.83 (0.39), 766	−3.45 (0.72), 867	—	—	−4.60 (0.36), 22	−3.66 (0.10), 10
	Superior frontal gyrus, medial	−2.68 (0.30), 375	−3.21 (0.47), 868	−2.71 (0.27), 536	−3.11 (0.50), 929	—	—	—	—
	Superior frontal gyrus, orbital part	−2.92 (0.45), 636	−2.62 (0.22), 153	−3.03 (0.38), 727	−2.55 (0.40), 464	—	—	—	—
	Superior frontal gyrus, medial orbital	−2.85 (0.44), 330	−2.50 (0.15), 94	−3.30 (0.60), 383	−2.81 (0.47), 239	—	—	—	—
	Middle frontal gyrus	−2.61 (0.30), 636	−2.78 (0.38), 564	−3.03 (0.53), 1,482	−2.72 (0.37), 957	—	—	−4.47 (0.31), 8	−4.37 (0.64), 55
	Middle frontal gyrus orbital part	−2.95 (0.44), 118	−2.80 (0.27), 90	−2.75 (0.38), 177	−2.56 (0.30), 133	—	—	—	—
	Inferior frontal gyrus, opercular part	−2.76 (0.37), 325	−2.47 (0.12), 188	−3.29 (0.34), 515	−2.76 (0.40), 493	—	—	—	—
	Inferior frontal gyrus, triangular part	−2.70 (0.42), 209	−2.53 (0.17), 135	−3.00 (0.49), 562	−2.39 (0.23), 472	—	—	—	—
	Inferior frontal gyrus, orbital part	−3.09 (0.48), 665	−2.62 (0.20), 84	−3.18 (0.51), 642	−2.40 (0.22), 205	—	—	−4.32 (0.12), 5	—
	Supplementary motor area	−2.51 (0.18), 26	−3.01 (0.47), 268	−2.71 (0.30), 273	−2.93 (0.47), 541	—	—	—	—
	Paracentral lobule	−2.22 (NA), 1	−2.47 (0.11), 21	−2.52 (0.17), 104	−2.58 (0.33), 338	—	—	—	—
	Gyrus rectus	−3.06 (0.42), 360	−2.85 (0.23), 91	−3.48 (0.58), 344	−3.01 (0.74), 235	—	—	—	—
	Olfactory cortex	−2.75 (0.33), 92	−2.67 (0.20), 38	−2.75 (0.36), 65	−2.76 (0.39), 55	—	—	—	—
Temporal lobe	Superior temporal gyrus	−3.19 (0.47), 1,680	−2.96 (0.43), 1,136	−3.75 (0.48), 1,787	−3.25 (0.46), 1,403	—	—	—	—
	Heschl gyrus	−2.83 (0.27), 250	−3.39 (0.26), 271	−3.32 (0.32), 251	−3.89 (0.27), 271	—	—	—	—
	Middle temporal gyrus	−2.87 (0.38), 1,617	−2.71 (0.33), 960	−3.55 (0.59), 1,663	−2.94 (0.42), 1,520	—	—	—	—
	Inferior temporal gyrus	−2.91 (0.44), 828	−2.72 (0.35), 174	−3.33 (0.58), 898	−2.57 (0.35), 446	—	—	—	—
Parietal lobe	Superior parietal gyrus	−2.51 (0.20), 129	−2.48 (0.12), 126	−2.61 (0.29), 328	−2.69 (0.46), 502	—	—	−4.59 (0.34), 60	−4.27 (0.50), 287
	Inferior parietal, but supramarginal and angular gyri	−2.57 (0.25), 111	−2.67 (0.25), 334	−2.95 (0.42), 287	−2.95 (0.47), 628	—	—	—	—
	Angular gyrus	−2.57 (0.22), 302	−3.10 (0.36), 583	−3.11 (0.47), 450	−3.39 (0.65), 623	—	—	−4.40 (0.26), 10	—
	Supramarginal gyrus	−3.31 (0.49), 526	−2.98 (0.47), 537	−3.78 (0.72), 776	−3.50 (0.38), 564	—	—	—	—
	Precuneus	−2.57 (0.33), 512	−2.73 (0.33), 481	−2.70 (0.40), 949	−2.88 (0.50), 1,518	—	—	−4.18 (NA), 1	−3.94 (0.25), 31
Occipital lobe	Superior occipital gyrus	−2.53 (0.26), 73	−2.39 (0.07), 112	−2.74 (0.31), 275	−2.78 (0.41), 396	—	—	—	—
	Middle occipital gyrus	−2.76 (0.28), 733	−2.53 (0.16), 506	−3.33 (0.55), 1,031	−2.75 (0.35), 1,490	—	—	—	—
	Inferior occipital gyrus	−2.39 (0.19), 7	−2.60 (0.22), 122	−2.73 (0.34), 111	−3.04 (0.28), 495	—	—	—	—
	Cuneus	−2.73 (0.44), 365	−2.51 (0.13), 127	−3.05 (0.51), 868	−3.00 (0.53), 961	—	—	—	—
	Calcarine fissure and surrounding cortex	−2.58 (0.20), 64	−2.49 (0.17), 59	−2.75 (0.36), 372	−2.82 (0.48), 897	—	—	—	—
	Lingual gyrus	−2.61 (0.31), 459	−2.72 (0.40), 259	−2.97 (0.51), 582	−3.04 (0.53), 902	—	—	—	—
	Fusiform gyrus	−3.54 (0.61), 883	−2.75 (0.32), 678	−3.50 (0.82), 1,000	−3.03 (0.42), 977	—	—	—	—
Limbic lobe	Temporal pole: Superior temporal gyrus	−3.07 (0.41), 422	−2.78 (0.28), 101	−3.15 (0.58), 434	−2.62 (0.38), 110	—	—	—	−3.61 (0.04), 5
	Temporal pole: Middle temporal gyrus	−2.89 (0.26), 112	—	−3.29 (0.43), 128	—	—	—	—	—
	Anterior cingulate and paracingulate gyri	−2.59 (0.25), 629	−2.97 (0.48), 144	−2.91 (0.36), 806	−2.78 (0.61), 911	—	—	—	—
	Median cingulate and paracingulate gyri	−2.77 (0.38), 408	−2.84 (0.46), 453	−2.96 (0.45), 902	−3.00 (0.55), 215	—	—	—	—
	Posterior cingulate gyrus	−2.53 (0.24), 102	−2.68 (0.25), 35	−2.61 (0.28), 217	−2.49 (0.37), 259	—	—	—	—
	Parahippocampal gyrus	−3.15 (0.59), 892	−2.78 (0.41), 174	−3.16 (0.63), 652	−2.79 (0.51), 461	—	—	—	—
Insular lobe	Insula	−3.02 (0.43), 840	−2.69 (0.31), 134	−3.22 (0.53), 942	−2.74 (0.53), 524	—	—	—	−3.68 (0.05), 2

*Note:* Correlation between the Expanded Disability Status Scale (EDSS) and the cortical thickness (CTh) of significantly associated cortical regions in all multiple sclerosis (MS) patients as well as separately in relapsing remitting MS (RRMS), secondary progressive MS (SPMS), and primary progressive MS (PPMS). Results represent the correlation strength shown in the form of *t*‐values extracted from our linear mixed effect models after correction with the false discovery rate approach for multiple comparisons set at *q* < 0.05. Lower (or more negative) *t*‐values reflect a stronger negative correlation. All results are reported in the form: mean *t*‐value (*SD*), number of vertexes in the respective region.

Abbreviation: NA, not applicable.

#### 3.5.2 RRMS

In the RRMS group, the log(EDSS) was not associated with the average CTh, after correcting for age and disease duration. However, log(EDSS) changes were negatively correlated with the CTh changes over time in large, extended bilateral cortical regions (*q* < 0.05) (Figure 5). These results are also shown in detail in Table 4.

#### 3.5.3 SPMS

In the SPMS group, the log(EDSS) was not associated with the CTh, after correcting for age (Figure 5).

#### 3.5.4 PPMS

In the PPMS group, the log(EDSS) was not correlated with the average CTh. However, log(EDSS) changes were negatively correlated with the CTh changes over time in small clusters, predominantly in the bilateral superior parietal gyri, the left precentral gyrus, the left middle frontal gyrus, and the left postcentral gyrus (*q* < 0.05). Results are also displayed in detail in Table 4 and Figure 5.

### 3.6 Association of CTh with the T25fwt, PASAT, and SDMT

In the whole cohort as well as in all MS subgroup analyses, the T25fwt and PASAT were not associated with the CTh. SDMT, only analyzed for the time span between the third and sixth follow‐up year was also not associated with the CTh (corrected for the same factors described above). However, as reported by the LMER analyses the PASAT and SDMT were shown to significantly improve over time in our MS patients (for the whole cohort: PASAT^2^ *B* = 48.01 ± 8.03/year, *p* < .001; SDMT *B* = 0.66 ± 0.17/year, *p* < .001), whereas this improvement in neuropsychological scores did not significantly differ between different MS subtypes (Table 1).

## 4 DISCUSSION

This is the first longitudinal study examining the relationship of CTh in a vertex‐wise manner with clinical‐ and lesion load measurements in a large cohort of different MS phenotypes over 6 years. Our work demonstrated similar temporospatial cortical changes over different disease subtypes, which —however— were related to disease progression in a disease‐type‐specific manner. We also showed an association of T2LV and CTh, although the effect of T2LV changes to longitudinal CTh changes was shown to be only marginal.

Our study demonstrated a significant CTh reduction over time in large prefrontal, frontal, parietal, and temporal cortical areas in all MS patients. These results are similar to a large cross‐sectional multicenter study comparing RRMS patients and healthy controls (Narayana et al., 2012). Therefore, it can be hypothesized that the observed atrophy demonstrated in these large cortical areas may represent a disease‐specific effect rather than the impact of aging, although our study could not confirm this hypothesis due to the absence of healthy controls. Similar results were shown in separate analysis for RRMS and SPMS patients, whereas no significant CTh reduction over time was shown in the PPMS. The latter finding should be considered with caution, since the sample size of our PPMS group was rather small (*n* = 12) and therefore this analysis may have lacked in power. This may be also supported by the findings of a recent large longitudinal volumetric study showing significant cortical atrophy in this group (Eshaghi et al., 2018).

In our patients, a clear effect of aging on CTh was demonstrated, which is in line with previous cross‐sectional and longitudinal studies of healthy individuals (Fjell et al., 2015; Thambisetty et al., 2010). In particular —as also seen in Figure 2, older patients were found to have reduced frontotemporal CTh as well as an accelerated cortical thinning in large cortical areas involving the prefrontal cortex, parieto‐occipital regions, and the superior temporal gyri. Aging‐related patterns of CTh have been shown to be driven by both genetic factors (Matsushita et al., 2015) and functional relationships of converging regions (Fjell et al., 2015).

Our analysis also revealed a correlation between CTh and disease duration with diffuse cortical thinning being apparent in later stages of the disease. The reported association between the loss of cortical GM and increasing disease duration is independent of normal aging, since disease duration was added after age in our LMER models. However, the rate of CTh reduction over time was not a function of disease duration, suggesting a steady cortical thinning throughout the course of the disease in patients during the monitoring time of our study.

As opposed to previous cross‐sectional studies and one recent large‐scale longitudinal volumetric studies of cortical GM in MS (Eshaghi et al., 2018; Fisher et al., 2008; Roosendaal et al., 2011), CTh did not differ between our RRMS, SPMS and PPMS groups, while age and disease duration possibly took up most of the CTh between‐group variance. Our results did not confirm the accelerated reduction of temporal cortical gray matter observed by Eshagi et al. in 2018. The discrepancy of those results may lie in the different methodological approaches of these studies, since the current study performed an analysis of the cortical shape or cortical thickness, whereas previous studies evaluated the cortical volume in different cortical areas. On the other side, our study included significantly less patients compared to the work done by Eshagi et al. in 2018, so that the power of our study might have been inadequate to reveal the aforementioned differences. In addition, differences of the two cohorts with regard to the disease modifying therapies of the enrolled patients could have also contributed to the discrepancy of the respective results. Finally, it could be hypothesized that differences in the age of MS patients of the two studies could also have been responsible for this discrepancy, since RRMS and especially SPMS patients were older in the current study compared to the longitudinal study conducted by Eshagi et al. in 2018.

Our work demonstrated a correlation of widespread CTh reduction with larger whole brain T2LV. This is in line with previous cross‐sectional studies suggesting that focal inflammatory events in the WM may—at least partially—“drive” cortical atrophy (Bergsland et al., 2015; Bodini et al., 2009; Henry et al., 2009). However, whole brain T2LV did not contribute to the temporal evolution of CTh over 6 years, suggesting that focal inflammatory events do not lead to an immediate loss of cortical GM. Similarly, the annualized relapse rate was not associated with CTh in RRMS. Further, exploration of a potential effect of the regional T2LV changes over time on CTh reduction over time revealed a correlation of the left temporal and left occipital T2LV with cortical changes over time, which is limited in the neighboring and contralateral corresponding GM. Thus, it is possible that WM lesions only produce a limited focal effect in the surrounding and anatomically connected cortex (e.g., through forceps major) as a result of intralesional axonal loss and following Wallerian degeneration. However, our results support that GM volume may be a process independent of focal WM pathology and MS relapses, at least to a certain extent. Nevertheless, a connection between T2LV changes over time or relapses with a longer‐term neurodegenerative process beyond the time span of our study affecting cortical gray matter cannot be excluded.

Temporal CTh changes were associated with the EDSS changes over 6 years in the whole cohort of MS patients. In particular, a large effect over cortical areas extending primarily in the bilateral prefrontal, frontal and temporoparietal regions was seen. In order to investigate the cortical changes responsible for disability progression in different MS groups, we also performed a separate analysis for our RRMS, SPMS, and PPMS patients. Herewith, it was revealed that the observed correlation between CTh and EDSS changes over time was driven primarily by the RRMS patients. Interestingly, in the SPMS patients no association was found between the EDSS and cortical changes. Moreover, in the PPMS patients the correlation between EDSS and CTh changes over time was found only in small clusters over the bilateral superior parietal gyri, the left precentral gyrus, and the left postcentral gyrus. These results suggest that the clinical impact of cortical changes is much more pronounced in RRMS than SPMS and PPMS patients. This also indicates a clear dissociation regarding the impact of the patients' CTh changes to disability in RRMS and progressive patients, especially since the CTh did not differ between groups. This finding is also in line with a previous study showing that other structures such as the spinal cord correlate better with progression of physical disability than brain metrics in progressive MS patients (Tsagkas et al., 2018; Tsagkas et al., 2019).

Surprisingly T25fwt, PASAT and SDMT did not correlate with the CTh neither in the whole cohort nor in the different MS‐subtypes. Concerning the T25fwt, it could be hypothesized that the large between‐patient variability could partly be responsible for the lack of association with CTh changes, even with motor‐related cortical areas. Moreover, other structures such as the spinal cord have been also shown to be better explanatory variables for T25fwt compared to brain metrics (Tsagkas et al., 2018; Tsagkas et al., 2019). Furthermore, in contrast to previous literature (Calabrese et al., 2009; Steenwijk et al., 2016), cognitive performance—as measured by PASAT and SDMT—in our cohort was not associated with CTh. However, a paradoxic significant improvement of those scores was evident in our patients in both scores, which may be attributed to a learning effect through repetition. This is in line with a number of studies including healthy controls and MS patients showing improved cognitive performance through practice or repetitive testing, even when testing was performed with relatively long intervals between follow‐up, similarly to our study (Baird, Tombaugh, & Francis, 2007; Bartels, Wegrzyn, Wiedl, Ackermann, & Ehrenreich, 2010; Basso, Bornstein, & Lang, 1999; Johnen et al., 2019; Roar, Illes, & Sejbaek, 2016).

There are some limitations of our study that have to be mentioned. We involved data of a big cohort of MS patients acquired retrospectively. As a consequence, some patients were lost to follow‐up during the study, leading to incomplete datasets and potential bias. However, all three examined MS subgroups (RRMS, SPMS, and PPMS) were monitored for a similar time period and thus represented in homogeneous fashion over our 6‐year follow up. Furthermore, the lack of a representative control group did not allow us to assess the CTh changes of MS compared to healthy subjects. Despite that, the correlation between CTh and clinical outcomes is independent of this limitation. Moreover, our study examined patients from a single center, which were scanned on a single MRI scanner. While this eliminated potential methodological issues arising from the utilization of different MR machines, due to the variability of the disease the acquisition of our data within a single center may somehow limit the generalizability of our results in other MS populations. However, the relatively large sample size of the investigated cohort and the long follow‐up period could have mitigated this issue. Another limitation of our work could refer to the different sample size between disease subtypes, which may have influenced our results. Notably, the PPMS group included a rather small number of patients; thus, interpretation and generalizability of these results for other PPMS populations should be done with caution. In addition, the majority of MS patients in our cohort (180 out of 243 patients) were classified as RRMS; therefore, it cannot be excluded that the contrast in the results between RRMS and progressive MS (especially concerning the correlation of CTh with the EDSS) is to be accounted for by the different MS subtype sample sizes in our cohort. Nevertheless, in our cohort, 64% of patients were treated with disease‐modifying including primarily first‐line injectables (60%). While injectables also show an effect on brain gray matter atrophy, based on previous studies, we believe that this effect is rather negligible (Favaretto, Lazzarotto, Margoni, Poggiali, & Gallo, 2018). During the collection of data in this study, there was no approved treatment for PPMS, so that no patient received treatment in this patient group. However, the distribution of disease modifying agents in our RRMS and SPMS patients did not significantly differ. Finally, it has to be noted that we did not examine the association between cortical lesions and CTh over time, since the sensitivity of T2‐ and PD‐weighted sequences for cortical lesions is known to be very low.

## 5 CONCLUSION

In conclusion, our study demonstrated a more prominent diffuse CTh reduction with increasing lesion load. However, only a marginal focal effect of regional T2LV changes to CTh changes over time was shown in neighboring and anatomically connected cortical areas, thus suggesting that GM atrophy progresses —at least partially— independent from focal inflammatory events. MS‐subgroups did not differ in terms of CTh. However, a clear dissociation in the correlation between CTh and EDSS changes over time between RRMS and SPMS patients was shown. Based on this finding we can hypothesize, that other CNS structures such as the spinal cord may be more relevant with regard to disability progression in SPMS patients.

## CONFLICT OF INTEREST

Charidimos Tsagkas, Chakravarty M. Mallar, Amann Michael have no disclosures. Naegelin Yvonne: Her employer, the University Hospital Basel received payments for lecturing from Celgene GmbH and Teva Pharma AG that were exclusively used for research support, not related to this study. K. Parmar: Her institution (University Hospital Basel) received speakers' honoraria from Novartis and ExceMED and travel support by Novartis Switzerland. Laura Gaetano was a temporary employee of Novartis AG and she is currently an employee of F. Hoffmann‐La Roche (her current institution was not involved in this project at any time). Athina Papadopoulou has received speaker‐fee from Sanofi‐Genzyme and travel support from Bayer AG, Teva, Roche and ECTRIMS. Her research was/is being supported by the University of Basel, the Swiss Multiple Sclerosis Society, the Swiss National Science Foundation and the “Stiftung zur Förderung der gastroenterologischen und allgemeinen klinischen Forschung sowie der medizinischen Bildauswertung”. J. Wuerfel: CEO of MIAC AG, Basel, Switzerland; speaker honoraria (Bayer, Biogen, Novartis, Teva); advisory boards and research grants (Biogen, Novartis); supported by the German Ministry of Science (BMBF/KKNMS) and German Ministry of Economy (BMWi). Ludwig Kappos' institution (University Hospital Basel) has received research support and payments that were used exclusively for research support for Dr Kappos' activities as principal investigator and member or chai r of planning and steering committees or advisory boards in trials sponsored by Actelion, Addex, Almirall, Bayer HealthCare, Celgene, CLC Behring, Genentech, GeNeuro, Genzyme, Merck Serono, Mitsubishi Pharma, Novartis, Octapharma, Ono, Pfizer, Receptos, F. Hoffmann‐La Roche, Sanofi‐Aventis, Santhera, Siemens, Teva, UCB, and XenoPort; license fees for Neurostatus 4 products; research grants from the Swiss Multiple Sclerosis Society, the Swiss National Research Foundation, the European Union, and the Roche Research Foundation. The current (DKD Helios Klinik Wiesbaden) or previous (University Hospital Basel) institutions of Till Sprenger have received payments for speaking or consultation from: Biogen Idec, Eli Lilly, Allergan, Actelion, ATI, Mitsubishi Pharma, Novartis, Genzyme, and Teva. Dr. Sprenger received research grants from the Swiss MS Society, Novartis Pharmaceuticals Switzerland, EFIC‐Grünenthal grant, and Swiss National Science foundation. Stefano Magon has received research support from Swiss Multiple Sclerosis Society, Swiss National Science Foundation, University of Basel and Stiftung zur Förderung der gastroenterologischen und allgemeinen klinischen Forschung sowie der medizinischen Bildauswertung University Hospital Basel. He also received travel support from Biogen and Genzyme.

## Data Availability

The data that support the findings of this study are available from the corresponding author upon reasonable request.
